# The Rash That Will Not Go Away

**DOI:** 10.1177/00099228261459939

**Published:** 2026-06-30

**Authors:** Peter Newman, Kushal Patel, Judith Rowen, Geetha Radhakrishnan, Donna Mendez

**Affiliations:** 1 Department of Pediatrics, The University of Texas Medical Branch, Galveston, USA; 2 Department of Emergency Medicine, The University of Texas Medical Branch, Galveston, USA

**Educational Objectives**

- The diagnosis of a chronic scaly crusted rash can be challenging. This patient’s case will highlight the clinical course of the disease and assist health care professionals in recognizing the diagnosis.
- The crusted scabies rash is characterized by large, crusted lesions, generalized scales, thick hyperkeratosis, and may involve the majority of total body surface area. Treatment includes topical permethrin in combination with oral ivermectin. Without adequate treatment, secondary infections can develop leading to bacteremia and sepsis in up to 8.8% of patients and should initially be treated for staphylococcal and streptococcal infections.

## Case Report

An 18-year-old male with Down syndrome due to Trisomy 21 and autism presented with complaints of a diffuse pruritic rash, that progressively worsened over the course of 1 year. The patient’s mother recalled the rash initially began around the bilateral eyebrows and hairline and progressively spread to the other parts of face, trunk, and upper extremities. He had been seen prior to admission twice and treated with oral prednisone and had mild improvement of symptoms. After the steroids were completed, the rash quickly returned in a more severe form, and he was then diagnosed with psoriasis and was prescribed topical steroids, which he did not tolerate due to pain during application of the ointment. Per the patient’s mother, he had been brought to the pediatric emergency department at this time because she was concerned that he had stopped eating and drinking, likely due to the pain caused by cheilosis and was admitted to the hospital. The patient had substantial weight loss of 30 pounds over the prior 5 months. The patient was admitted for the severity of his rash and pain management, which prevented him from eating and drinking.

His medical history included a diagnosis of Down syndrome and autism. His surgical history included cardiac surgery at the age of 5 months due to (unknown) cardiac defect, orchidopexy at 2 years, and tonsillectomy at 5 years of life. The patient lived with his parents in a mobile home. At baseline, the patient could not speak or chew solid foods, but was able to walk with assistance, and understood simple commands. Patient was able to eat pureed food on his own before developing the rash on his hands. His parents also had symptoms of a pruritic erythematous rash.

On physical exam, he was alert and active. His temperature was 101.4°F, blood pressure was 102/66, heart rate was 94, respiratory rate was 16, and oxygen saturations 98 on room air. His weight was 41 kg and his height was 157 cm, putting him on the <1 percentile on Centers for Disease Control and Prevention (CDC) (boys, 2-20 years) weight-for-age and <1 percentile based on CDC (boys, 2-20 years) stature-for-age. His scalp had multiple erythematous papules in linear fashion along with multiple crusted lesions, his eyes had crusted lesions involving upper and lower eyelids bilaterally. His ear exam included erythematous papules and crusted lesions involving both ears, nose exam with multiple erythematous papules, and neck with multiple erythematous papules in linear fashion along with multiple crusted lesions. His lung exam was clear to auscultation, heart exam with regular rate and rhythm, no murmur, and abdomen exam with normal bowel sounds, soft, non-distended, no hepatosplenomegaly or masses. His skin exam revealed multiple erythematous papules in linear fashion along with multiple crusted lesions all over the body and burrows seen between fingers.

This case study did not require institutional review board’s approval. There was no conflict of interest and no funding for this case report.

## Discussion

### Final Diagnosis

Our patient with a history of Down syndrome and autism had a diagnosis of crusted scabies (CS) with bacteremia caused by methicillin-sensitive *Staphylococcal aureus* (MSSA) and *Serratia marcescen*s. A child with CS has an increased risk of secondary bacterial infections that can lead to bacteremia and sepsis.^1^ *Serratia marcescens* is a rare cause of bacteremia in children with CS. The differential diagnosis of CS included erythrodermic psoriasis, erythrodermic seborrheic dermatitis, and pemphigus foliaceus. Because the child lived in a mobile home, he could have had an allergic mold reaction or irritant contact dermatitis due to formaldehyde. Mobile homes are prone to mold due to high moisture and lack of ventilation. Mobile homes also have formaldehyde-treated plywood and adhesives that can cause irritant contact dermatitis.^2^

### Hospital Course

**Figures 1, 2, 3.**
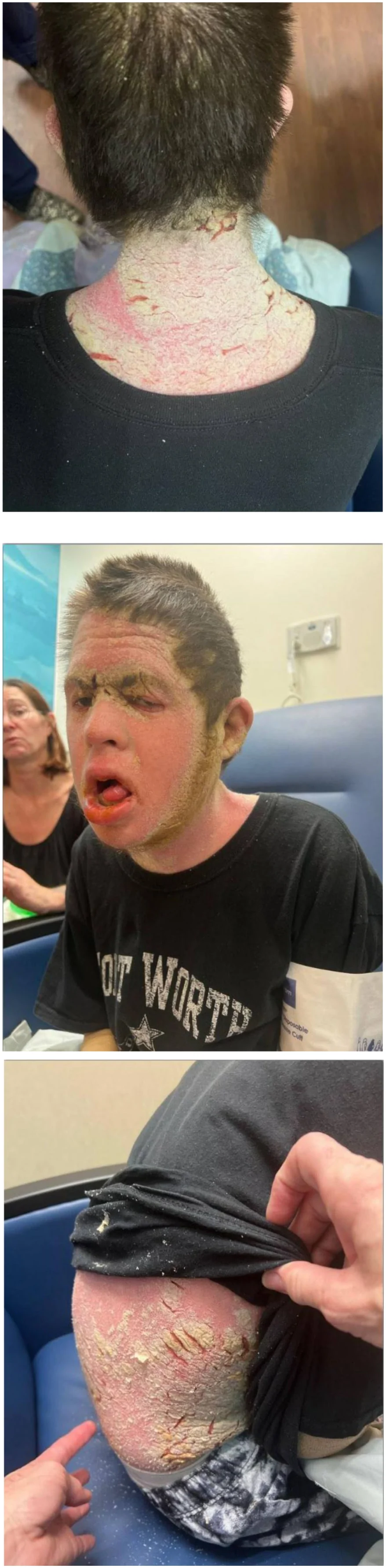
Before admitted to hospital.

Upon admission to hospital on the pediatric floor, he was non-toxic appearing, alert but had a temperature of 101.4°F and had erythematous papules and crusted lesions over 80% of his body (Figures 1-3). Shortly after admission, blood cultures were reported to be positive for MSSA and the patient was started on doxycycline, and nafcillin for the MSSA. The repeat blood culture 4 days after admission was positive for *S. marcescen*s, so that the antibiotics were switched to vancomycin and cefepime, but then changed to nafcillin and ceftriaxone as recommended by pediatric infectious disease physicians. Ultimately, the blood cultures were negative, and he was prescribed 10 more days of oral levofloxacin on discharge. The patient’s blood work revealed a slightly elevated white blood cell of 13.53 (normal 4.5-13.50) and absolute neutrophil count of 11.5 (normal 15.0-10.3). His inflammatory markers were elevated, C-reactive protein 3.5 (normal <0.08), procalcitonin 0.80 (normal <0.07) yet erythrocyte sedimentation rate normal at 20 (normal 2-30). There were no laboratory results significant for an immune or chronic granulomatous disease.

**Figure 4,5.**
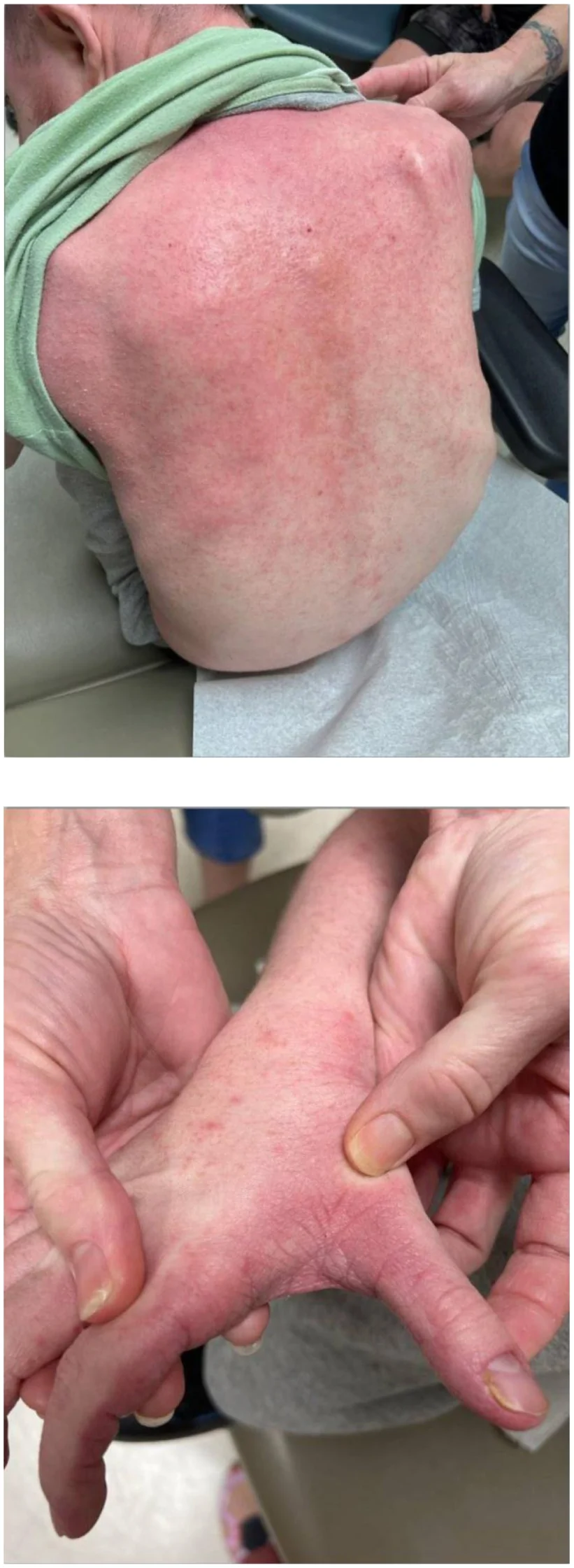
After treatment.

Dermatology was consulted on hospital day 1, with a 4 mm biopsy performed from his neck and direct immunofluorescence obtained. Mineral oil scraping was performed and showed scabies mites, eggs, and feces. The patient was started on oral ivermectin to treat the scabies. In addition, he was also started on triamcinolone ointment and IV Benadryl for symptom control. The treatment with permethrin ointment was started once the patient’s skin condition improved to the point that it was not painful to apply (Figures 4 and 5). He was seen again at outpatient dermatology clinic, who noted dramatic improvement of symptoms, and prescribed another round of permethrin cream to the patient and family members.

## Discussion

Crusted scabies, previously known as “Norwegian” scabies, is a rare and severe form of scabies less frequent in children compared with adults.^3^ It is characterized by large, crusted lesions, generalized scales, and thick hyperkeratosis.^4^ While hands and feet are most affected, the most severe cases have near total body surface involvement.^5^ There is a delay of diagnosis per the literature from 2 to 18 months.^6,7^ Diagnosis can be based on clinical presentation, history, and physical exam, and can be confirmed with skin scrapings or skin biopsy.^8^ Without adequate treatment, secondary infections can lead to bacteremia or sepsis.^1^ Our patient had MSSA and *S. marcescens* bacteremia. Staphylococcal and streptococcal infections^9^ are the most common organisms with bacteremia/sepsis being reported in 8.8% of the cases.^10^ Treatment of MSSA bacteremia consists of a beta-lactam agent such as nafcillin, oxacillin, flucloxacillin, or cefazolin,^11^ with our patient receiving nafcillin. Empiric treatment for methicillin-resistant *Staphylococcus aureus* (MRSA), which is another common bacteria causing bacteremia consists of vancomycin.^12^ *Serratia marcescens* is a rare secondary infection from scabies with only one report of a wound culture being positive with *S. marcescens*,^13^ but there are no reports on *S. marcescens* bacteremia. *Serratia marcescens* bacteremia is usually treated with fluoroquinolones, aminoglycosides, trimethoprim-sulfamethoxazole, piperacillin-tazobactam, third- and fourth-generation cephalosporins, aztreonam, and carbapenems^14^ with our patient receiving a third-generation cephalosporins.

Our patient had Down syndrome that has been associated with CS^8,10,15^ and felt to be due to an immune dysfunction^16^ or lack of response to a pruritic rash.^10,16^ Most individuals with healthy immune systems can mount effective responses against the scabies mite.^17^ Mechanical debridement of mites and eggs via scratching is an effective mechanism to limit proliferation but patients with cognitive impairment who are unable to interpret the itch or unable to scratch are at increased risk of developing CS.^8^ In addition, Down syndrome patients are thought to have an immunodeficiency with a significant decrease in switched memory B and T cells.^15,18^ Also, there is an elevated baseline cytokine level prior to any induced inflammation.^19^

Systemic and topical corticosteroids have been associated with CS. Locally applied corticosteroids (especially fluorinated) alter the skin’s immune system, with the inflammatory and cellular response reduced.^19^ In addition, systemic steroids can decrease the pruritic symptoms and immune response allowing the mites to proliferate and develop into CS. From the literature, children with CS, have a prevalence of prior corticosteroid use of 75%.^20^ Our patient received both systemic and topical steroids.

This is the first report of a pediatric patient with autism having CS. Autism by itself has been associated with dysfunction in B, T and NK cells and increased production of cytokines and auto-antibodies.^21,22^ In Down syndrome and autism, there is an elevated baseline level of cytokines, but how exactly it is related to immune dysfunction is speculative.^21,23^ One theory is that there is a cytokine storm when the body is induced with more inflammation such as with a virus that leads to a decrease in antibacterial defenses.^22^ Instead of a virus perhaps scabies induced more inflammation, which led to this cytokine storm with decreased antibacterial defenses leading to bacteremia in our patient.

The treatment for CS is more intense than classic scabies. An approach to the patient with CS is to initiate combination treatment with topical permethrin and oral ivermectin and treatment of the pruritus. This combination of topical scabicide (permethrin) and oral ivermectin has been shown to be effective.^24-27^ The use of lindane is contraindicated for CS due to the risk for toxicity.^28^ For the pruritus associated with the rash can be treated with oral antihistamines such as hydroxyzine and diphenhydramine. Opinions vary on the use of topical corticosteroid therapy. A complication of CS is secondary bacterial infection where treatment is geared toward staphylococcal and streptococcal infections. Hospitalization is recommended for secondary bacterial infections and may be indicated for moderate to severe pain^29^ or severe rash.^30^

## Conclusion and Lessons Learned

The diagnosis of CS can be challenging, especially in patients who are unable to communicate their symptoms such as in children with Down syndrome and autism. In addition, Down syndrome and autism patients have an immune deficiency, which may lead to a more severe form of scabies, CS. Although steroids are often used in dermatological conditions, the steroids provided to this patient only provided temporary improvement and may have exacerbated the condition. In this case, early referral to a dermatologist may have led to diagnosis and appropriate therapy, thus limiting the course of the disease. Without adequate treatment, secondary infections can develop leading to bacteremia and sepsis in up to 8.8% of patients and should initially be treated with antibiotics against streptococcus and staphylococcus.

## Author Contributions

P Newman contributed to the concept and drafting, and revision of manuscript. K Patel, J Rowan, and G Radhakrishnan contributed to the concept, drafting, and revision of manuscript. D Mendez contributed to critical revision of the manuscript. All authors take responsibility for the paper.
